# IL-1**β**–dependent extravasation of preexisting lung-restricted autoantibodies during lung transplantation activates complement and mediates primary graft dysfunction

**DOI:** 10.1172/JCI157975

**Published:** 2022-10-17

**Authors:** Wenbin Yang, Emily Jeong Cerier, Félix L. Núñez-Santana, Qiang Wu, Yuanqing Yan, Chitaru Kurihara, Xianpeng Liu, Anjana Yeldandi, Nigar Khurram, Diego Avella-Patino, Haiying Sun, G.R. Scott Budinger, Daniel Kreisel, Thalachallour Mohanakumar, Emilia Lecuona, Ankit Bharat

**Affiliations:** 1 Division of Thoracic Surgery,; 2 Department of Pathology, and; 3 Pulmonary and Critical Care Medicine, Feinberg School of Medicine, Northwestern University, Chicago, Illinois, USA.; 4 Departments of Surgery, Pathology & Immunology, Washington University, St. Louis, Missouri, USA.; 5 Norton Thoracic Institute, St. Joseph’s Hospital and Medical Center, Phoenix, Arizona, USA.

**Keywords:** Transplantation, Innate immunity, Organ transplantation

## Abstract

Preexisting lung-restricted autoantibodies (LRAs) are associated with a higher incidence of primary graft dysfunction (PGD), although it remains unclear whether LRAs can drive its pathogenesis. In syngeneic murine left lung transplant recipients, preexisting LRAs worsened graft dysfunction, which was evident by impaired gas exchange, increased pulmonary edema, and activation of damage-associated pathways in lung epithelial cells. LRA-mediated injury was distinct from ischemia-reperfusion injury since deletion of donor nonclassical monocytes and host neutrophils could not prevent graft dysfunction in LRA-pretreated recipients. Whole LRA IgG molecules were necessary for lung injury, which was mediated by the classical and alternative complement pathways and reversed by complement inhibition. However, deletion of Fc receptors in donor macrophages or mannose-binding lectin in recipient mice failed to rescue lung function. LRA-mediated injury was localized to the transplanted lung and dependent on IL-1β–mediated permeabilization of pulmonary vascular endothelium, which allowed extravasation of antibodies. Genetic deletion or pharmacological inhibition of IL-1R in the donor lungs prevented LRA-induced graft injury. In humans, preexisting LRAs were an independent risk factor for severe PGD and could be treated with plasmapheresis and complement blockade. We conclude that preexisting LRAs can compound ischemia-reperfusion injury to worsen PGD for which complement inhibition may be effective.

## Graphical abstract

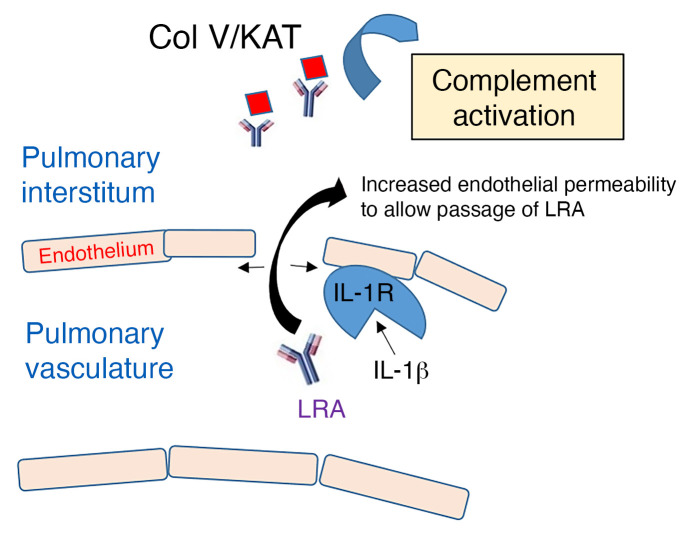

## Introduction

Primary graft dysfunction (PGD) affects over 50% of recipients following lung transplantation and has emerged as the principal risk factor for both short-term mortality as well as long-term graft loss from chronic rejection (1–4). Current empiric therapies to treat PGD are largely ineffective and have the attendant risks of immunosuppression. While ischemia-reperfusion injury remains its predominant cause, the highly variable incidence of PGD suggests additional etiologies for its pathogenesis. A significant percentage of patients undergoing lung transplantation have preexisting immunoglobulin G (subtype IgG2) autoantibodies against lung-restricted self-antigens collagen type V (COLV) and K-α1 tubulin (KAT) (5, 6). The presence of these lung-restricted autoantibodies (LRAs) in the recipients is associated with an increased risk of PGD following both human (6) and murine (5, 7–9) lung transplantation. However, the molecular events by which LRAs promote PGD and their relationship to transplant-inherent ischemia-reperfusion injury remain unknown. Understanding of the pathogenesis of PGD in recipients with underlying LRAs will enable the development of selective therapies to improve posttransplant outcomes.

Investigators have suggested a mechanism to explain the production of LRAs in patients with chronic lung diseases (10). The deletion of self-reactive T lymphocytes against lung self-antigens by the thymus is incomplete, and escaped self-reactive T lymphocytes are dynamically suppressed through peripheral regulatory T cells (11). Depletion of regulatory T cells in response to environmental challenge promotes the expansion of self-reactive T lymphocytes and development of LRAs (10). The lung self-antigens are nonpolymorphic and are normally sequestered from the immune system, as they serve as scaffolds for the structural proteins (12). However, during transplantation the self-antigens might be revealed as the structural proteins are cleaved, for example through the activation of matrix metalloproteinases, allowing the preexisting LRAs to bind and promote downstream immune activation (13). These self-antigens are extravascular but the increased vascular permeability that occurs during ischemia-reperfusion injury could allow extravasation of the LRAs (14). We found that LRAs compound ischemia-reperfusion injury through the activation of complement, resulting in severe PGD. Our findings reveal pathways through which LRAs mediate lung allograft injury that can be targeted clinically.

## Results

### Intact LRAs promote primary graft dysfunction.

**Figure 1:**
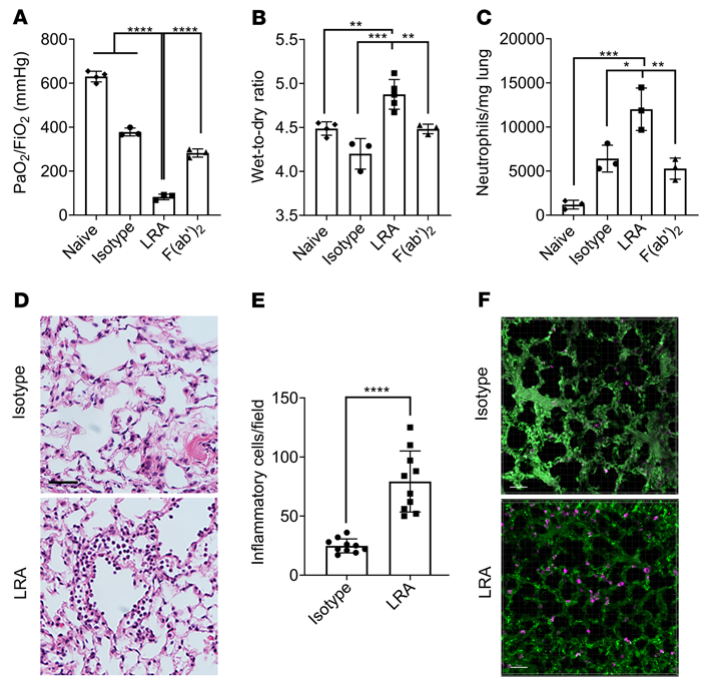
Preexisting whole lung-restricted antibodies (LRAs) against self-antigens induce primary graft dysfunction after syngeneic murine lung transplantation. Recipient mice received 150 μg each (i.v.) of isotype control, LRAs (anti–collagen type V plus anti–K-α1 tubulin), or LRA F(ab′)_2_ portion, 24 hours before and 1 hour after lung transplantation. (**A**) Twenty-four hours after transplantation, arterial blood oxygenation was analyzed after clamping the right hilum (*n =* 3–4). Lungs were also harvested for assessment of (**B**) pulmonary edema (*n =* 3–5) and (**C**) neutrophils (live CD45^+^SiglecF^–^CD11b^+^Ly6G^+^) (*n* = 3). (**D**) Histology showing capillaritis and alveolar edema in mice treated with LRAs but not isotype control antibodies. Scale bar: 20 μm. (**E**) Inflammatory cells (both polymorpho- and mononuclear) were counted in 10 high-power fields (×40) and averaged for each group. (**F**) Pictures from 2-photon microscopy showing LRA (magenta) deposition. Scale bar: 50 μm. Data are presented as mean ± SD. PaO_2_/FiO_2_, arterial oxygen pressure. Graphs in **A**–**C** were analyzed by 1-way ANOVA followed by Tukey’s post hoc test. Graph in **E** was analyzed by unpaired, 2-tailed Student’s *t* test. *P < 0.05; **P < 0.01; ***P < 0.001; ****P < 0.0001.

**Figure 2:**
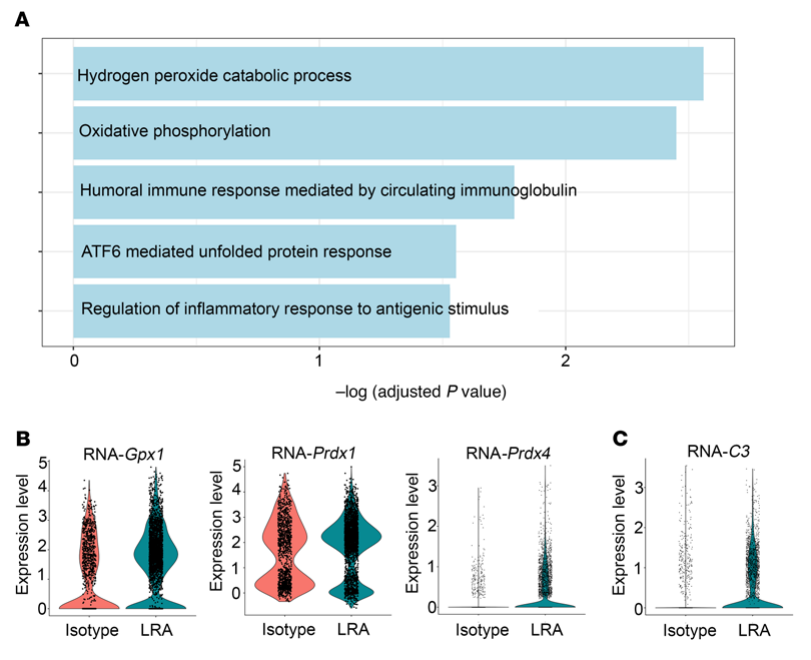
Differential expression analysis of single-cell RNA-seq data from isotype- or LRA-treated murine allograft after lung transplantation within epithelial cells. (**A**) Functional enrichment analysis with GO Biological Processes was performed with the significantly upregulated genes in epithelial cells from LRA-treated compared with isotype-treated murine lung allograft. (**B** and **C**) Violin plots of expression for select genes significantly upregulated in epithelial cells from LRA-treated compared with isotype-treated murine lung allograft.

LRAs belong to the IgG family of immunoglobulins (5, 6), which are composed of Fc and F(ab′)_2_ fragments. The F(ab′)_2_ fragment exhibits sequence variability and recognizes antigens, while the Fc fragment provides interaction sites for effector molecules such as complement and Fcγ receptors (FcγRs) (15). However, F(ab′)_2_ can also independently interact with FcγRs to mediate immunological effects (16). To determine the pathogenicity of LRAs, we injected IgG isotype, LRAs, or the cleaved F(ab′)_2_ fragment into recipient mice prior to transplantation. As expected, ischemia-reperfusion injury was inherent to this model and experienced by all animals, evident by decreased lung graft function in the recipients of IgG isotype control compared with naive lung (isotype PaO_2_/FiO_2_ ratio: 452.3 ± 63 mmHg; naive PaO_2_/FiO_2_ ratio: 621.9 ± 32 mmHg; *P <* 0.0001). Recipients injected with whole LRA IgG molecules showed significant worsening in lung graft function (PaO_2_/FiO_2_ ratio: 104.2 ± 41 mmHg; Figure 1A) as well as increased pulmonary edema (Figure 1B), while those injected with F(ab′)_2_ fragments demonstrated expected levels of lung graft dysfunction resulting from ischemia-reperfusion injury (Figure 1, A and B). There was increased neutrophil infiltration determined by flow cytometry in mice with whole LRA molecules (Figure 1C) along with capillaritis and inflammatory cells found on histological analysis (Figure 1, D and E). In addition, 2-photon imaging (Figure 1F, Supplemental Figure 1, and Supplemental Videos 1 and 2; supplemental material available online with this article; https://doi.org/10.1172/JCI157975DS1) showed increased LRA deposition in the allograft after lung transplantation. Unbiased transcriptomic analysis using single-cell RNA sequencing (RNA-seq) (Supplemental Figure 2) revealed activation of several damage-associated pathways (Figure 2A), including oxidative stress (reflected as increased expression of antioxidant proteins; Figure 2B) and complement (increased C3 expression; Figure 2C) in the epithelial cells during LRA-mediated lung graft injury.

### LRAs activate classical and alternative complement pathways to mediate lung graft injury.

**Figure 3:**
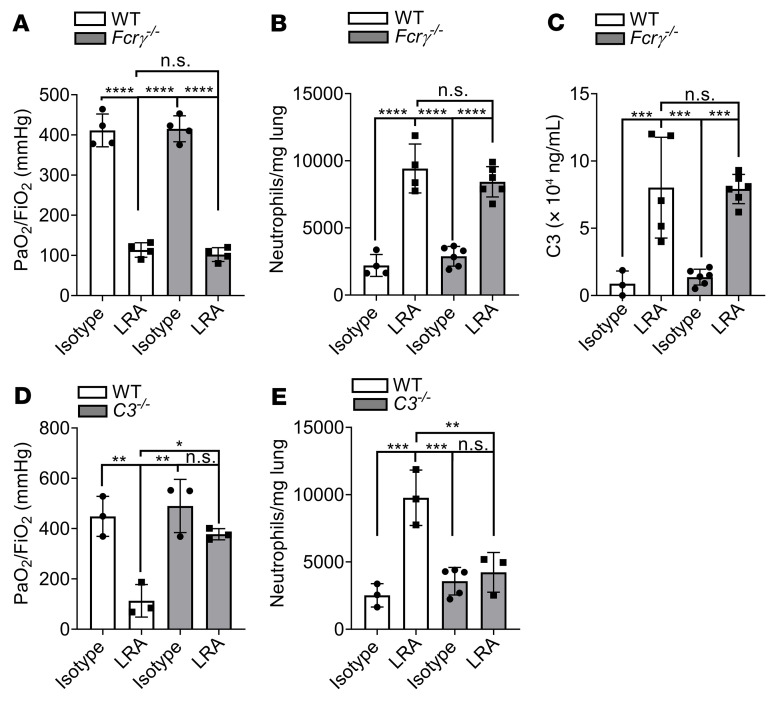
LRAs activate complement pathway to mediate lung graft injury. (**A**) WT or *Fcrγ^–/–^* recipients received 150 μg each (i.v.) of isotype control or LRAs (anti–collagen type V plus anti–K-α1 tubulin) 24 hours before and 1 hour after lung transplantation. Arterial blood oxygenation was analyzed after clamping the right hilum (*n =* 4). (**B**) Lungs treated as in **A** were also harvested for neutrophil quantification (live CD45^+^SiglecF^–^CD11b^+^Ly6G^+^) (*n* = 3). (**C**) WT or *Fcrγ^–/–^* recipient mice were treated as in **A**. Twenty-four hours after transplantation, bronchoalveolar lavage fluid (BALF) was obtained and C3 concentration measured by ELISA (*n =* 3–6). (**D**) WT or *C3^–/–^* recipient mice were treated as in **A**. Arterial blood oxygenation was analyzed after clamping the right hilum (*n =* 3). (**E**) Quantification of neutrophil recruitment into lungs in mice treated as in **A** (neutrophil gating: live CD45^+^SiglecF^–^CD11b^+^Ly6G^+^) (*n =* 3–5). Data are presented as mean ± SD. PaO_2_/FiO_2_, arterial oxygen pressure. Graphs were analyzed by 1-way ANOVA followed by Tukey’s post hoc test. *P < 0.05; **P < 0.01; ***P < 0.001; ****P < 0.0001; NS, not significant.

Since F(ab′)_2_ could not induce PGD, we reasoned that the Fc region was necessary for the pathogenic effects of LRAs. Accordingly, we investigated whether LRA-mediated lung injury occurred through FcγR or complement activation (17). Donor lungs from *Fcrγ*^–/–^ mice transplanted into syngeneic recipients pretreated with LRAs experienced severe lung injury, similar to those from wild-type donor lungs (Figure 3, A and B). We used these mice, as donors and not recipients as the predominant macrophages in the donor lungs at 24 hours are of donor origin (18). This led us to further hypothesize that LRAs activate complement to induce lung graft dysfunction.

**Figure 4:**
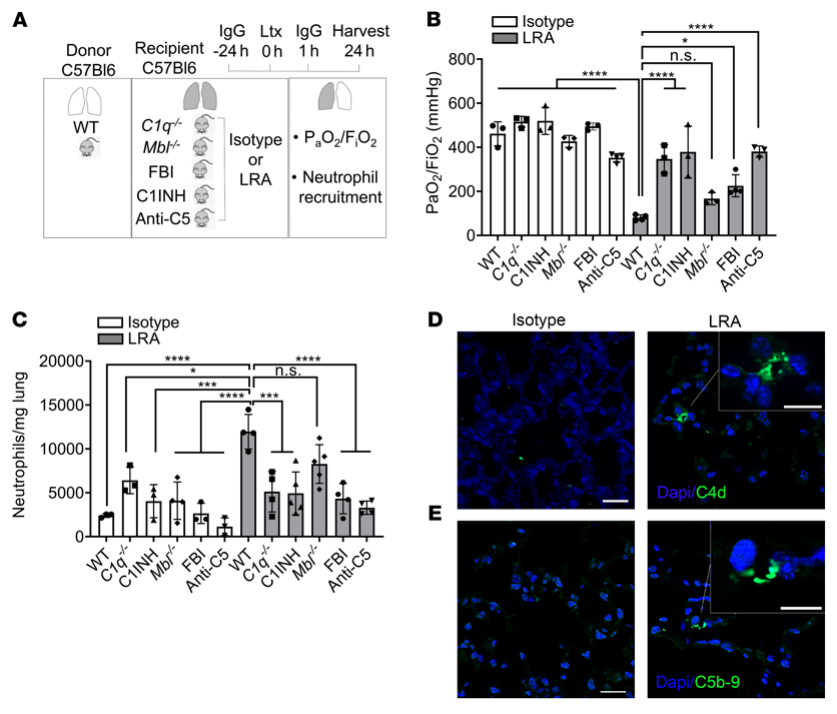
LRAs activate classical and alternative complement pathways to mediate lung graft injury. (**A**) Diagram depicting experiments shown in **B** and **C**. WT, *C1q^–/–^*, or *Mbl^–/–^* recipient mice received 150 μg each (i.v.) of isotype control or LRAs (anti–collagen type V plus anti–K-α1 tubulin) 24 hours before and 1 hour after lung transplantation (Ltx). For some experiments, WT recipient mice treated with antibodies as described received C1INH, LNP023 (Factor B inhibitor, FBI), or anti-C5 neutralizing antibody. (**B**) Arterial blood oxygenation from mice described in **A** was analyzed after clamping the right hilum (*n =* 3). (**C**) Quantification of neutrophil recruitment into lungs in mice treated as described in **A** (neutrophil gating: live CD45^+^SiglecF^–^CD11b^+^Ly6G^+^) (*n* = 3–5). (**D** and **E**) Immunocytochemistry for C4d (**D**) and C5b-9 (**E**) in allografts of LRA-treated mice (right) compared with isotype-treated mice (left). Data are presented as mean ± SD. PaO_2_/FiO_2_, arterial oxygen pressure. Graphs were analyzed by 1-way ANOVA followed by Tukey’s post hoc test. *P < 0.05; ***P < 0.001; ****P < 0.0001; NS, not significant. Scale bars: 20 μm and 10 μm (insets).

We first studied component C3, as it acts as a point of convergence for the different complement pathways. Indeed, C3 levels were elevated in the BAL of grafts from wild-type or *Fcrγ*^–/–^ recipients pretreated with LRAs but not in those that received isotype control (Figure 3C). Moreover, genetic deletion of C3 in recipient mice resulted in nearly complete protection against LRA-induced lung graft dysfunction (Figure 3, D and E). Next, we determined the specific complement pathway activated by LRAs using genetic deletion and pharmacological inhibition. We transplanted wild-type donor lungs into *C1q*^–/–^ (classical pathway), *Mbl*^–/–^ (mannose-binding lectin, lectin pathway), or FBI-treated (factor B inhibitor, alternative pathway) recipients. Each of these recipients also received LRAs or isotype control antibodies prior to the transplant. Preexisting LRAs induced severe lung graft dysfunction in *Mbl*^–/–^ mice, while the *C1q*^–/–^ and FBI-treated recipients were protected (Figure 4, B and C). Furthermore, treatment of recipient mice with a pharmacological C1 inhibitor (C1INH) resulted in protection of the transplanted lungs from LRA-induced injury as well as associated increases in neutrophil recruitment (Figure 4, B and C). Immunohistochemistry confirmed complement C4d deposition, a marker of classical pathway activation, in the lung grafts of LRA-treated recipients (Figure 4D). All 3 complement pathways can induce the formation of the membrane attack complex (MAC). MAC assembly requires the sequential and irreversible association of complement proteins C5b, C6, C7, C8, and C9 (19, 20). Immunohistochemical analysis showed MAC deposition in grafts from LRA-recipient mice (Figure 4E). Inhibiting activation of C5, and thus the generation of C5a and MAC, has shown great therapeutic benefit in complement-driven inflammatory diseases (21). Hence, we inhibited C5 by using a C5-blocking antibody and found prevention of LRA-induced graft dysfunction (Figure 4, B and C). Together, these data suggested that LRAs activate both the classical and alternative complement pathways to mediate lung graft injury.

### LRA-induced lung dysfunction is complementary but distinct from ischemia-reperfusion injury.

**Figure 5:**
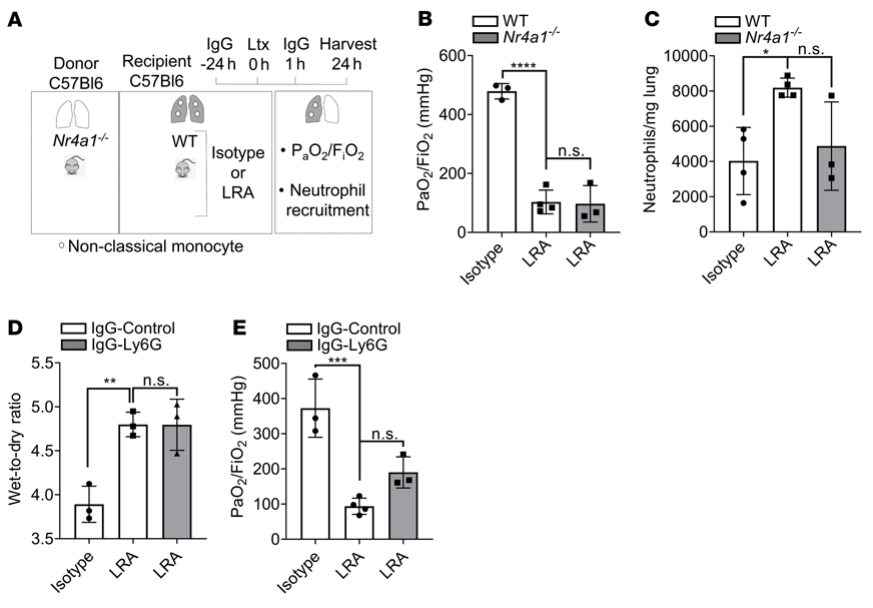
LRA-induced lung dysfunction is complementary to ischemia-reperfusion injury. (**A**) Diagram depicting experiment shown in **B** and **C**. WT recipient mice received 150 μg each (i.v.) of isotype control or LRAs (anti–collagen type V plus anti–K-α1 tubulin) 24 hours before and 1 hour after lung transplantation (Ltx) using donor *Nr4a1^–/–^* mice. (**B**) Arterial blood oxygenation from mice described in **A** was analyzed after clamping the right hilum (*n =* 3). (**C**) Quantification of neutrophil recruitment into lungs in mice treated as described in **A** (neutrophil gating: live CD45^+^SiglecF^–^CD11b^+^Ly6G^+^) (*n* = 3–4). (**D**) WT recipient mice treated as in **A** were injected (i.p.) with isotype or anti-Ly6G antibody 24 hours before lung transplantation and harvested for determination of pulmonary edema 24 hours after transplantation (*n =* 3). (**E**) WT recipient mice treated as described in **D**. Arterial blood oxygenation was analyzed after clamping the right hilum (*n =* 3–4). Data are presented as mean ± SD. PaO_2_/FiO_2_, arterial oxygen pressure. Graphs were analyzed by 1-way ANOVA followed by Tukey’s post hoc test. *P < 0.05; **P < 0.01; ***P < 0.001; ****P < 0.0001; NS, not significant.

We have previously reported that ischemia-reperfusion injury is inherent to lung transplantation and is dependent on the influx of host neutrophils into the transplanted lung through retained donor-origin pulmonary intravascular nonclassical monocytes (22, 23). To determine whether LRAs can independently induce lung graft dysfunction, we used lungs from murine *Nur77*^–/–^ (*Nr4a1*^–/–^) donors, which lack nonclassical monocytes and do not experience neutrophil-mediated ischemia-reperfusion injury, as we previously reported (23). We found that transplantation of nonclassical monocyte–deficient donor lungs into LRA-pretreated recipients resulted in severe lung graft dysfunction (Figure 5, B and C). Nevertheless, it was intriguing that lung grafts in the presence of donor nonclassical monocytes revealed increased neutrophil influx when LRAs were present in the recipients prior to transplantation (Figure 1C). To determine whether neutrophils contributed to the pathogenesis of LRA-induced lung graft injury, we depleted host neutrophils using anti-Ly6G antibodies, as previously described (18) (Supplemental Figure 3). Neutrophil depletion in mice with preexisting LRAs did not prevent posttransplant lung injury, as measured by pulmonary edema (Figure 5D and Supplemental Figure 3) and PaO_2_/FiO_2_ ratio (Figure 5E). Comparison of lung function between isotype and LRA (PaO_2_/FiO_2_ [mmHg] isotype: 372.6 ± 82 [*n =* 3]; LRA: 93.7 ± 23 [*n =* 4]; *P <* 0.001) and isotype-IgG-control and LRA-IgG-Ly6G (PaO_2_/FiO_2_ [mmHg] isotype: 434 ± 49 [*n =* 3]; LRA: 190 ± 44 [*n =* 3]; *P <* 0.01) showed no protection in graft injury. These data indicated that LRA-induced lung graft dysfunction is complementary to neutrophil-mediated ischemia-reperfusion injury and predominantly occurs through complement activation.

### IL-1β is necessary for vascular endothelial permeabilization and extravasation of LRAs.

**Figure 6:**
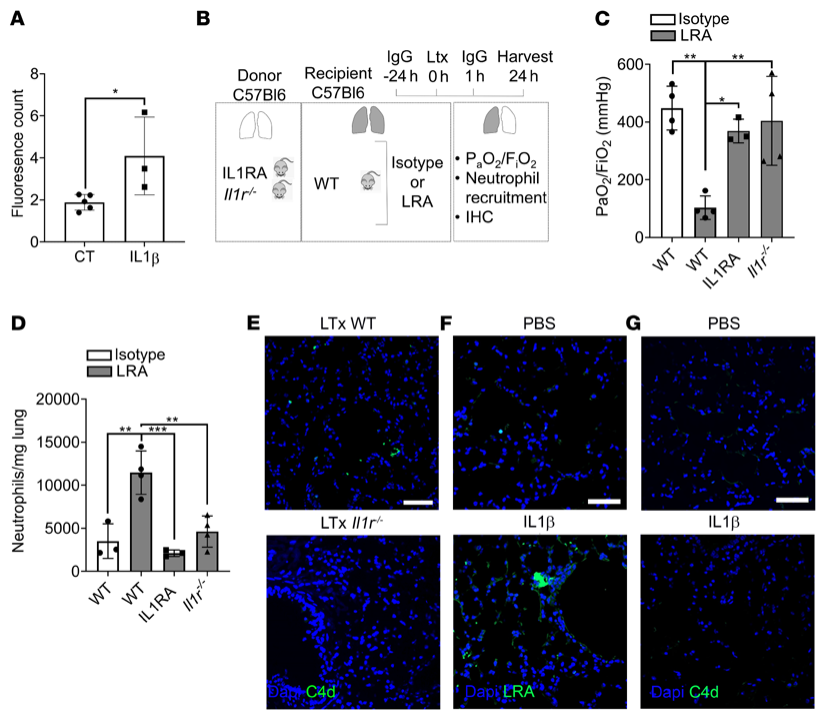
IL-1β is necessary to increase vascular endothelial permeability and extravasation of LRAs. (**A**) Mouse primary lung microvascular endothelial cells were seeded at 200,000 cells per insert and cultured until confluent. Monolayers were incubated for 24 hours in the absence (control, CT) or presence of 50 ng/mL IL-1β in growth medium. FITC-antibody permeability testing was performed as described in the Methods. (**B**) Diagram depicting experiment shown in **C** and **D**. WT recipient mice received 150 μg each (i.v.) of isotype control or LRAs (anti–collagen type V plus anti–K-α1 tubulin) 24 hours before and 1 hour after lung transplantation (Ltx). Donor lungs were from *Il1r^–/–^* mice or WT mice treated with IL-1RA 24 hours before and 1 hour after lung transplant. (**C**) Arterial blood oxygenation from mice described in **B** was analyzed after clamping the right hilum (*n =* 3). (**D**) Quantification of neutrophil recruitment into lungs in mice treated as described in **B** (neutrophil gating: live CD45^+^SiglecF^–^CD11b^+^Ly6G^+^) (*n* = 3–5). (**E**–**G**) Immunohistochemistry for C4d (**E** and **G**) and LRAs (**F**) in allografts of LRA-treated wild-type mice (upper) compared with LRA-treated *Il1r^–/–^* mice (lower) (**E**) or in lungs from PBS- or IL-1β–treated mice (**F** and **G**). Data are presented as mean ± SD. PaO_2_/FiO_2_, arterial oxygen pressure. Graphs were analyzed by 1-way ANOVA followed by Tukey’s post hoc test. *P < 0.05; **P < 0.01; ***P < 0.001. Scale bars: 20 μm.

The native lung of the LRA-treated recipients showed no histological evidence of injury. Additionally, there was no increase in neutrophils in the native lungs of LRA-treated recipients (neutrophils/mg lung isotype: 1685 ± 760; LRA: 1445 ± 1008). Lung-restricted antigens are present in the interstitial space (12) and, hence, we next hypothesized that extravasation of LRAs into the interstitial space, which might uniquely occur in the transplanted lung graft, was necessary for the activation of complement. We have previously shown that IL-1β can increase pulmonary vascular permeability and allow cellular extravasation (24). Hence, we tested whether release of IL-1β within the transplanted lung was necessary to permit extravasation of circulating LRAs and gain access to the antigens. We first treated primary pulmonary endothelial cells in vitro with exogenous IL-1β (50 ng/mL) and found that it significantly increased endothelial permeability and the passage of LRAs through the barrier (Figure 6A). We then transplanted wild-type, IL-1R antagonist–treated (IL-1RA–treated), or *Il1r^–/–^* lungs into wild-type LRA-treated syngeneic recipients. Donor lungs treated with IL-1RA or those from *Il1r^–/–^* mice showed preserved lung graft function (Figure 6C), decreased neutrophil influx (Figure 6D), and no C4d deposition (Figure 6E). There was no difference in graft function among the isotype and LRA group treated with IL-1RA or when *Il1r^–/–^* donor lung was used (PaO_2_/FiO_2_ [mmHg] IL-1RA–isotype: 515.7 ± 24 [*n =* 3]; *Il1r^–/–^*-isotype: 412 ± 141 [*n =* 3]; IL-1RA–LRA: 368 ± 41 [*n =* 3]; *Il1r^–/–^*-LRA: 404 ± 154 [*n =* 4]). To further investigate the role of IL-1β, we injected wild-type mice with IL-1β or PBS, 8 hours prior to injecting LRAs, and determined LRA deposition and complement activation. We found retention of LRAs (Figure 6F) with IL-1β pretreatment in naive lungs, similar to lung allografts (Supplemental Figure 1), but it was not associated with C4d deposition (Figure 6G), indicating that IL-1β is necessary for the extravasation of LRAs but not sufficient to trigger LRA-mediated complement activation.

### Preexisting LRAs are an independent risk of human PGD.

We prospectively analyzed LRAs in 56 patients undergoing lung transplantation. The cumulative prevalence of detectable preexisting anti-COLV and anti-KAT antibodies was 14.3% in this cohort. The demographic profile of the patients is presented in Table 1. In the study cohort, 4 (7.1%) patients developed grade 3 PGD: 2 (25%) with and 2 (4.5%) without preexisting LRAs (*P =* 0.09; Supplemental Table 1). Logistic regression modeling revealed that only preexisting LRAs were an independent predictor of grade 3 PGD after lung transplantation (Supplemental Table 2 and Table 2). In patients with preexisting LRAs, apostreperfusion biopsy was performed at 60 minutes. Both patients with preexisting LRAs who developed grade 3 PGD revealed histological features reminiscent of acute antibody-mediated rejection with complement (C4d) deposition (Supplemental Figure 4). These patients were treated with the complement inhibitor eculizumab, along with plasma exchange, and experienced resolution of lung allograft dysfunction (Supplemental Figure 4).

## Discussion

Neutrophil-mediated ischemia-reperfusion injury is suggested to be the predominant cause of PGD (1, 25). However, it is evident that the syndrome of PGD includes etiologies other than ischemia-reperfusion injury. Preexisting LRAs are present in a significant proportion of patients undergoing lung transplantation and are strongly associated with an increased risk of PGD (6). Studies in which right and left lungs from single donors were transplanted into different recipients with or without autoantibodies have further suggested their role in the pathogenesis of PGD (26). However, the mechanistic pathways through which LRAs contribute to PGD remain unclear. Importantly, the relationship between LRA-induced graft dysfunction and classical pathways that contribute to transplant-inherent ischemia-reperfusion injury remains unknown. Our current study identifies clinically actionable pathways through which preexisting LRAs add to ischemia-reperfusion injury to worsen PGD. Given that the LRAs caused severe lung graft injury even when neutrophil-mediated ischemia-reperfusion injury was mitigated, we postulate that treatment of LRA-induced lung graft injury could result in amelioration of PGD and improvement in posttransplant outcomes.

The complement system is an essential part of the innate immune system that has been associated with ischemia-reperfusion injury and transplantation (27). Indeed, PGD has been associated with complement deposition, although whether these transplant recipients had preexisting LRAs is unknown (28). Additionally, while preexisting LRAs have also been associated with complement deposition in lung grafts, whether they can activate complement remains unclear (26). The classical complement pathway depends on the binding of C1q protein to antibody attached to antigen, activating C1r and C1s, which cleave C4 and C2. The lectin complement pathway is activated when mannose-binding lectin (MBL) interacts with carbohydrate motifs on pathogens. This results in the activation of MBL-associated proteases, which also cleave C4 and C2. Hence, both classical and lectin pathways form C3 convertase after cleaving C4 and C2 and initiate the downstream proteins. In contrast, the alternative pathway is activated by spontaneous hydrolysis of C3, in the presence of Factors B and D, leading to the eventual formation of the C3 and C5 convertase. In the alternative pathway, properdin plays an important role as it stabilizes the protein. All 3 pathways culminate in the formation of effector compounds (29). Since deletion of MBL did not alter the graft injury in LRA-pretreated mice, we conclude that the lectin pathway is not involved. However, C1q and Factor B inhibition prevented LRA-induced lung graft dysfunction, suggesting a role for classical and alternative complement pathways in this model. Our findings also explain the immunological mechanisms behind the reported detection of complement proteins in patients with PGD (30). However, the differences with our study might be related to our focus on LRAs, where IgG glycosylation might have a less relevant role than C1q binding to the Fc portion of IgG. It is also noteworthy that while LRAs might activate host cellular immunity and phagocytosis by binding FcγRs on target cells, those did not contribute to the LRA-mediated graft dysfunction since *Fcrγ^–/–^* donor lungs experienced similar injury to that of wild-type grafts. Our findings have immediate clinical implications since complement inhibitors are FDA approved and commercially available.

Unlike histocompatibility antigens, the cognate self-antigens for the LRAs are nonpolymorphic and conserved (31). Ischemia-reperfusion can reveal epitopes of self-antigens that typically serve as structural lung proteins (32), possibly through the activation of matrix metalloproteinases (5, 33–35). Exposure of the self-antigens can then promote the binding with LRAs (7). We have also previously shown that donor-origin nonclassical monocytes retained in the pulmonary vasculature initiate the pathogenesis of ischemia-reperfusion injury through the recruitment of recipient neutrophils (22, 23). Simultaneously, donor nonclassical monocytes activate donor alveolar macrophages, which secrete monocyte chemoattractant protein-1, necessary for the mobilization of classical monocytes from the recipient spleen (18, 24). Upon migration to the transplanted lung allograft, the recipient classical monocytes increase the pulmonary vasculature permeability through the release of IL-1β, allowing the neutrophils to extravasate and initiate the pathogenesis of ischemia-reperfusion injury (24). We found that increased endothelial permeability mediated by IL-1β was also necessary for the extravasation of LRAs into the extravascular space and initiation of LRA-induced lung graft injury. Nevertheless, LRAs caused lung injury even when donor nonclassical monocytes were depleted, which results in incomplete recruitment of recipient classical monocytes (18, 24). This may suggest alternative sources of IL-1β production or the ability of even low levels of IL-1β to cause sufficient endothelial permeability to allow LRA extravasation. Interestingly, administration of IL-1β into wild-type naive mice induced extravasation of LRAs into the lung interstitium without activating complement (Figure 6). It is known that the lung self-antigens, COLV and KAT, are normally sequestered, typically forming scaffolds for larger structural proteins such as collagen type I (36). As such, the immunogenic epitopes of these self-antigens are not accessible to the immune system or the extravasated antibodies in resting mice. When we administered IL-1β to permeabilize the vascular endothelium, the circulating LRAs extravasated into the interstitium but were unable to activate complement, as they did not bind to their cognate antigen. In contrast, during ischemia-reperfusion injury, there is activation of catalytic enzymes such as matrix metalloproteinases (37), which can cleave these self-antigens, enabling the formation of LRA-antigen immune complex and activation of complement. Hence, these findings suggest the need for both IL-1β and ischemia-reperfusion injury to activate the complement and induce C4d deposition (38, 39). Together, these data suggest that while the mechanisms of LRA-induced injury are distinct from ischemia-reperfusion injury, the molecular and immunological changes during ischemia-reperfusion are necessary for the pathogenicity of LRAs. Our findings also suggest inhibition of IL-1β or the IL-1β receptor as a therapy to prevent both ischemia-reperfusion and LRA-associated injury. Notably, canakinumab and anakinra, agents that block IL-1β and the IL-1β receptor, respectively, have relatively benign short-term safety profiles and have received FDA approval for other indications.

Our study has limitations. First, we cannot determine whether prophylaxis or desensitization against LRAs is beneficial in our model. Second, it is difficult to distinguish between antibody-mediated rejection and ischemia-reperfusion injury clinically based on histologic features alone. Histological features such as alveolar edema, neutrophil infiltration, and capillaritis can be observed in both ischemia-reperfusion injury as well as antibody-mediated rejection, although the presence of complement deposition may favor the former (6, 40–43). Future prospective studies, potentially including transcriptional profiling (Figure 2), are necessary to distinguish pathological and molecular features to better characterize these disease states and enable delivery of therapies in the correct clinical context. Recently, exosomes containing lung-restricted antigens have been detected during PGD in patients containing LRAs and could potentially be tested as a biomarker (44). Given the potential for complications associated with severe immunosuppression, prophylactic use of complement inhibitors in patients with LRAs is not scientifically justified. Instead, the approach in our center is to use histology and complement staining, when clinically feasible, to guide the use of complement inhibitors (45). Nevertheless, this should be prospectively validated in clinical trials. Third, while we tested the known LRAs associated with PGD in patients and causally linked to lung graft injury in animal models, we acknowledge that it is unclear whether there are other immunogenic lung-specific antigens. Future studies incorporating contemporary techniques in proteomics and mass spectroscopy can potentially identify these proteins as well as their immunogenic epitopes. Fourth, while only 2 patients developed features reminiscent of antibody-mediated rejection, their response to treatment combined with our preclinical data provides the foundation for a prospective clinical trial. Fifth, while we found that neutrophils are recruited during LRA-induced lung injury above the expected levels during ischemia-reperfusion injury, the mechanisms remain unknown. Donor nonclassical monocytes play a dominant role in recruiting host neutrophils through the secretion of chemokines and it is possible that the LRA-antigen complex may enhance donor nonclassical monocytes by binding CD16.

In conclusion, we found that, in lung transplants, IL-1β increased pulmonary endothelial permeability to allow the extravasation of LRAs. Binding of LRAs to cognate self-antigens in the pulmonary interstitium and ischemia-reperfusion caused the activation of classical and alternative complement pathways, which contributed to the immunopathogenesis of PGD, independent of neutrophil-mediated ischemia-reperfusion injury. C5-inhibiting antibodies as well as Factor B inhibition mitigated LRA-associated lung graft dysfunction in humans and mice.

## Methods

### Mice and procedures

Male wild-type C57BL/6J (B6), *Fcrγ^–/–^*, *Il1r^–/–^*, *C1qa^–/–^*, *C3^–/–^*, *Mbl^–/–^*, and *Nr4a^–/–^* mice were obtained from The Jackson Laboratory. IL-1RA (0.2 ng/g body weight i.v.; Sigma-Aldrich) was used for IL-1β–IL-1R antagonism. C1INH (0.4 U/g body weight, Berinert, CSL Behring) and LNP023 (Factor B inhibitor, 30 μg/g body weight, Adooq Bioscience) were injected i.v. in recipients 24 hours before and 1 hour after lung transplantation. IL-1β (10 μg/kg, i.v.; Thermo Fisher Scientific) was injected 8 hours prior to LRA injection. Neutrophils were depleted by using anti-Ly6G antibody (12.5 mg/kg body weight; Bio X Cell, clone 1A8). Complement C5 was inhibited by using a C5-blocking antibody (100 μg/mouse, 1 hour before lung transplant; provided by Alexion Pharmaceuticals). Control mice were treated with the same amounts of IgG isotype control antibody (Bio X Cell). All mice were maintained in a specific pathogen–free facility at the Center for Comparative Medicine at Northwestern University and used for the described experiments at the age of 9–14 weeks and between 24 and 28 g of body weight.

### Mouse lung transplant.

Orthotropic murine left lung transplantation was performed as previously described (46). Briefly, donor mouse was anesthetized with a mixture of xylazine (10 mg/kg) and ketamine (100 mg/kg). Donor lungs were flushed through the pulmonary artery with 3 mL of saline solution and the heart-lung block was excised and kept in cooled (4°C) preservative solution. The bronchus, pulmonary vein, and artery were dissected and prepared for anastomosis. A customized cuff made with a Teflon intravenous catheter was applied to the vascular structures and fixated with a 10-0 nylon ligature. After placement of a microvessel clip on the bronchus to avoid airway infiltration with preservative solution, the graft was stored at 4°C for a period of 90 to 120 minutes of cold ischemic time prior to implantation. The recipient mouse received subcutaneous buprenorphine (0.1 mg/kg) 30 minutes prior to the thoracic surgical incision and every 6 hours as needed after the transplant procedure. The recipient mouse was intubated and a left-sided thoracotomy was performed within the third intercostal space. The recipient’s native lung was gently clamped and pulled out of the thoracic cavity. The space between the artery, the vein, and the bronchus was dissected separately. The artery and vein were temporarily occluded using 8-0 nylon ligatures. The anastomoses were completed by fixating each cuff with 10-0 nylon ligatures. The 8-0 ligatures were released (first vein, then artery) and the lung inflated. The chest incision was closed and recipients separated from the ventilator when spontaneous respiration resumed. No antibiotics or immunosuppressive agents were used postoperatively in any group. LRAs were administered i.v. in recipient mouse: 150 μg each (anti-COLV and anti-KAT) or 300 μg isotype rabbit IgG (Thermo Fisher Scientific) 24 hours before and 1 hour after lung transplantation.

### Antibodies against KAT and COLV

Rabbit polyclonal IgG antibodies against KAT and COLV were produced against KAT and COLV proteins as previously described (47). Purified antibodies were endotoxin free by limulus amebocyte lysate assay. F(ab′)_2_ fragments of IgG antibodies against KAT and COLV were prepared with a Pierce F(ab′)_2_ Preparation Kit, as described by the manufacturer (Thermo Fisher Scientific). For 2-photon microscopy, anti-COLV antibody was conjugated to R-PE using a PE/R-Phycoerythrin Conjugation Kit - Lightning-Link (Abcam, ab102918).

### Arterial blood gases

Arterial blood gases were measured using a fraction of inspired oxygen of 100% after the right pulmonary hilum was clamped for 5 minutes (8). Blood was collected by left ventricular puncture.

### Wet to dry weight ratio

The transplanted left lung was harvested after reperfusion at defined time points, weighed, and then placed at 54°C until a stable dry weight was achieved. The ratio of wet weight to dry weight was then calculated as an indicator of pulmonary edema.

### Flow cytometry

Mouse lung was digested and single-cell suspensions were prepared as previously described (46). Cell suspensions underwent red blood cell lysis using Pharm Lyse buffer (BD Biosciences). Live/dead staining was performed in protein-free solution (HBSS) using fixable viability dye eFluor 506 (eBioscience), followed by incubation with FcR-blocking reagent (Miltenyi Biotec). Antibodies utilized for murine cell staining included rat anti–mouse CD45–FITC (BioLegend, 30-F11), rat anti–mouse Ly6C–eFluor450 (eBiosciences, HK1.4), rat anti–mouse I-A/I-E–PerCPCy5.5 (BioLegend, M5/114.15.2), rat anti–mouse CD45–APC (BioLegend, 30-F11), rat anti–mouse Ly6G–Alexa Fluor 700 (BioLegend, 1A8), rat anti–mouse NK1.1–Alexa Fluor 700 (BD, PK136), rat anti–mouse CD11b–APCCy7 (BioLegend, M1/70), rat anti–mouse CD64–PE (BioLegend, X54-5/7.1), rat anti–mouse SiglecF–PECF594 (BD, E50-2440), and rat anti–mouse CD11c–PECy7 (BD, HL3). For neutrophil quantification, 123Count eBeads (Invitrogen) were added. Flow analysis of fixed samples was done on a BD FACSymphony A5-Laser Analyzer at the Northwestern University Robert H. Lurie Comprehensive Cancer Center Flow Cytometry Core facility. Acquired data were analyzed with FlowJo v10.8 (FlowJo).

### Histology

Tissue sections were stained with hematoxylin and eosin and analyzed blindly. Images were obtained on a Nikon Eclipse microscope or Olympus DP-71, and morphometric analysis was performed using Nikon Elements software. Analysis was performed on 10 different areas of the lungs, and at least 10 high-powered fields were analyzed in each area.

### Single-cell RNA-seq

Single-cell suspensions from 24-hour posttransplant allografts from isotype- or LRA-treated mice were prepared as described previously (18). Allograft was removed and digested with 3 mL Dispase (Corning) with DNase I (Sigma-Aldrich), and gently teased using forceps into small (1–2 mm) fragments followed by incubation at room temperature with gentle agitation for 30 minutes. The resulting suspension (in DMEM + 5% FBS) was passed through a 70 μm cell strainer, erythrocytes were lysed, and filtered through 40 μm cell strainers. Cells were counted using AO/PI and Cellometer K2 (Nexcelom), and cell viability exceeded 90%. Single-cell 3′ RNA-seq libraries were prepared using Chromium Single Cell V2 Reagent Kit and Controller (10× Genomics). Libraries were assessed for quality (TapeStation 4200, Agilent) and then sequenced on a NextSeq 500 or HiSeq 4000 instrument (Illumina). Initial data processing was performed using the Cell Ranger version 6.0 pipeline (10× Genomics), and reads were mapped to mm10 version of the mouse genome, Ensemble build 105. Ambient RNA contamination was removed by the SoupX package (48). Doublets were evaluated by scrublet and removed (49). Downstream single-cell RNA-seq analysis was performed using Seurat Package version 4.0 following the standard workflow posted on the Satija lab website (https://satijalab.org/seurat/) (50). Cells with unique feature counts less than 200 or over 7500 were removed. Cells with RNA counts less than 400 or over 40,000 were removed. Cells having greater than 10% mitochondrial counts were removed. The data were normalized by the “LogNormalize” method and variable features were selected by “vst” method. Reciprocal principal component analysis was used for the data integration. Cell types were identified using both manual annotations based on positive markers from FindAllMarkers function in the Seurat pipeline and a supervised annotation tool, SingleR (51). The RNA-seq data can be found in the NCBI Gene Expression Omnibus repository (GEO GSE211501).

### Two-photon intravital lung microscopy.

Two-photon intravital lung microscopy imaging was performed using a Nikon A1R-MP+ multiphoton microscope system with a Coherent Chameleon Vision titanium sapphire laser. As previously described (18), mice were anesthetized with an i.p. injection of ketamine (80 mg/kg) and xylazine (10 mg/kg), intubated orotracheally, and ventilated with room air at a rate of 120 breaths/minute with a tidal volume of 0.5 mL. A left thoracotomy was performed to expose the left lung, and the lung was imaged using a custom-built chamber maintained at 37°C. Vetbond was used to attach the lung tissue to the bottom of the cover glass without direct pressure on the exposed lung. For time-lapse imaging of location and extent of anti-COLV–PE deposition in lungs, we averaged 37 video-rate frames (0.5 seconds per slice) during the acquisition to match the ventilator rate and minimize movement artifacts. To visualize blood vessels, 25 μL of FITC-dextran (2 × 10^6^ Da, 5 mg/mL, Thermo Fisher Scientific) in 25 μL of PBS was injected i.v. 5 minutes prior to imaging. For each mouse, images of 512 × 512 μm in the *x* and *y* dimensions were acquired for 5 minutes prior to and for 15 minutes following injection of anti-COLV–PE (1 mg/kg i.v.) using a water immersion lens (Apo LWD 25× 1.10W DIC N2) at an excitation wavelength tuned at 1000 nm. Images were processed and analyzed using Nikon NIS-Elements NIS.ai and Bitplane Imaris software for background/fluorophore spillover subtraction and fluorescence quantification of deposited fluorophores. Data were transferred and plotted in GraphPad Prism 9.0 (Sun Microsystems) for analysis and creation of graphs.

### Bronchoalveolar lavage fluid

The right pulmonary hilum was clamped, and left lung bronchoalveolar lavage fluid (BALF) was obtained by instilling lung airways 2 times with 0.5 mL PBS. BALF was centrifuged and the supernatant was used for complement C3 determination.

### ELISA

Mouse complement C3 ELISAs were performed using commercially available kits accordingly to the manufacturer’s instructions (Abcam). To determine autoantibodies in patients’ serum, LABScreen autoantibody kits (LSAUT1-3, One Lambda) was used according to the manufacturer’s instructions.

### Immunohistochemistry

Mice were anesthetized with pentobarbital sodium (50 mg/kg, i.p.) and lung tissues were excised and fixed with 10% neutral buffered formalin. Paraffin-embedded sections were incubated with rabbit anti-C4d IgG (1:200, overnight at 4°C; Hycult Biotech, HP8033) or rabbit anti–C5b-9 IgG (Bioss Antibodies, bs-2673R) and then successively reacted with donkey anti–rabbit IgG H&L (Alexa Fluor 488) (1:500, 1 hour at room temperature; Abcam, ab150073). For LRA deposition, paraffin-embedded sections were incubated directly with with donkey anti–rabbit IgG H&L (Alexa Fluor 488) as above. Nuclei were stained with Hoechst 33342 (MilliporeSigma). Confocal images were acquired using a Zeiss Axio Imager Z2 with ApoTome.2 microscope equipped with Axiocam 503 Mono, X-Cite 120 LED Boost System and Zen 2.3 software (Carl Zeiss).

### In vitro endothelial cell permeability

Lung primary microvascular endothelial cells (Cell Biologics, C57-6011) were grown in phenol red–free endothelial cell medium (ScienCell), seeded in collagen-coated inserts, and permeability was determined using a vascular permeability assay kit according to the manufacturer’s instructions (Millipore). FITC-conjugated anti-COLV antibody, labeled using a FITC Conjugation Kit (Fast) - Lightning-Link (Abcam), was added to wells in the absence or presence of 50 ng/mL recombinant mouse IL-1β (Thermo Fisher Scientific) and fluorescence was red with a plate reader with filters appropriate for 485 nm and 535 nm excitation and emission, respectively.

### Definition of primary graft dysfunction

Patients with no evidence of pulmonary edema on chest x-ray were considered grade 0. Absence of invasive mechanical ventilation was graded according to the PaO_2_/FiO_2_ ratio, using methods similar to those receiving mechanical ventilation. If PaO_2_ was not available for calculation of a PaO_2_/FiO_2_ ratio, then an oxygen saturation/FiO_2_ ratio was used for calculations. Grade 3: PaO_2_/FiO_2_ ratio < 200. The worst PaO_2_/FiO_2_ ratio within 72 hours after lung transplantation was used. Use of extracorporeal lung support with bilateral pulmonary edema on chest x-ray image was considered grade 3.

### Immunosuppressive therapy

All lung transplant recipients received standardized immunosuppressive therapy. Methylprednisolone 500 mg i.v. intraoperatively and basiliximab 20 mg i.v. intraoperatively and postoperative day (POD) 4 were given as induction immunosuppressive therapy.

Maintenance immunosuppression was conducted as follows: Prednisone 0.5 mg/kg p.o. daily from POD 1, mycophenolate mofetil 1000 mg i.v. bid from POD 1, and tacrolimus 0.015 mg/kg total daily dose sublingual, targeting from 8 to 12 ng/mL.

Patients with LRAs that developed PGD underwent treatment for antibody-mediated rejection using i.v. methylprednisolone (1000 mg) for 3 days, and daily plasmapheresis for 3 days followed by i.v. immunoglobulin (1 mg/kg) and eculizumab (1200 mg, 900 mg, and 600 mg on days 1, 2, and 3).

### Statistics

#### Murine.

Mouse data analysis was performed using Prism 8 (GraphPad Software). Results are expressed as mean ± SD and the *n* values for each data set are provided in the figure legends. Statistical significance was assessed by 2-tailed Student’s *t* test or 1-way ANOVA followed by Tukey’s post hoc test. A *P* value of less than 0.05 was considered significant.

#### Human.

Incidence of grade 3 primary graft dysfunction after lung transplantation was compared between the LRA-positive and LRA-negative groups. Continuous variables were compared using a 2-tailed Student’s *t* test and are reported as mean ± SD. Categorical variables were compared using Fisher’s exact test and are reported as numbers (percentages). *P* values of less than 0.05 were accepted as statistically significant. A logistic regression model was used to derive odds ratios and 95% CIs. To build our models, we performed a univariate analysis and included all predictors if the test had a *P* value of 0.2 or less. To assess the overall goodness of fit, we used Gronnesby’s and Borgan’s tests. Statistical analyses were performed using EZR (Saitama Medical Center, Jichi Medical University, Saitama, Japan), which is a graphical user interface for R (The R Foundation for Statistical Computing, Vienna, Austria). It is a modified version of R commander designed to add statistical functions frequently used in biostatistics.

### Study approval

#### Murine.

All procedures were approved by the Institutional Animal Care and Use Committee (IS00010493) at Northwestern University. Animals received humane care in compliance with the NIH *Guide for the Care and Use of Laboratory Animals* (National Academies Press, 2011) and the Principles of Laboratory Animal Care formulated by the National Society for Medical Research.

#### Human.

Patient data were collected retrospectively using the electronic medical records and stored in a database. A total of 56 adult patients undergoing lung transplantation at our institution were prospectively tested for antibodies. This study was approved by The Northwestern University Institutional Review Board (STU00207250). However, the need for patient consent for data collection was waived by the IRB as this was a retrospective study.

## Author contributions

WY, EL, and DK contributed to conceptualization, study design, methodology, data collection, validation, formal analysis, and manuscript writing. EJC, FLNS, YY, NK, and CK contributed to formal analysis and methodology. QW, AY, and HS conducted experiments. XL, DAP, GRSB, and TM contributed to formal analysis, visualization, and manuscript writing. AB contributed to conceptualization, methodology, validation, formal analysis, investigation, resources, data curation, writing, visualization, supervision, project administration, and funding acquisition.

## Supplementary Material

Supplemental data

Supplemental video 1

Supplemental video 2

## Figures and Tables

**Table 1:**
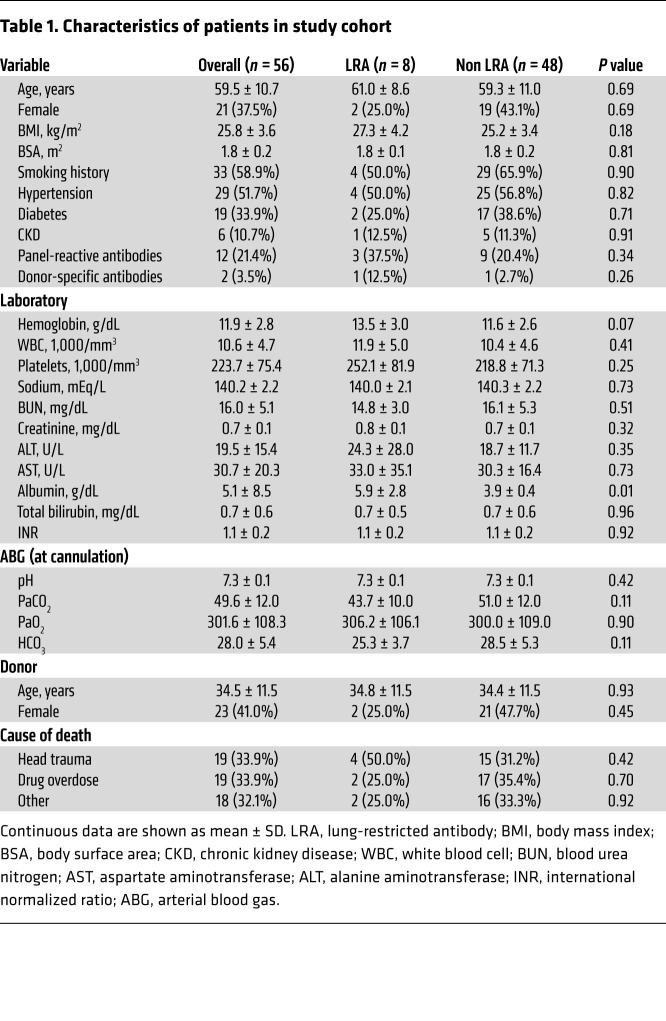
Characteristics of patients in study cohort

**Table 2:**
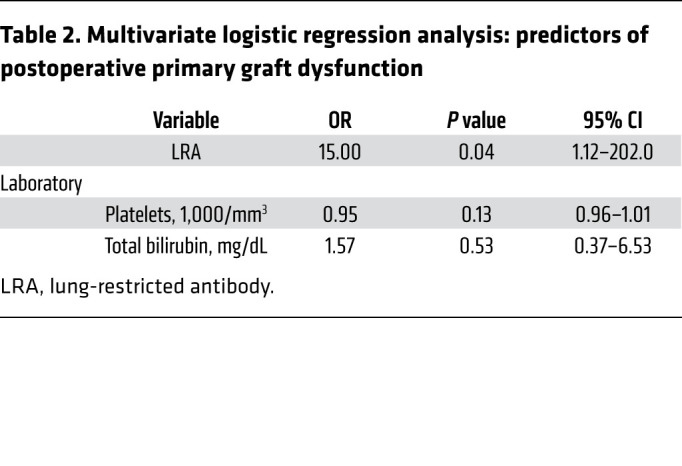
Multivariate logistic regression analysis: predictors of postoperative primary graft dysfunction

## Version 1 10/17/2022

Electronic publication
