# Supplementary material for: IL-1β–dependent extravasation of preexisting lung-restricted autoantibodies during lung transplantation activates complement and mediates primary graft dysfunction
Source: J Clin Invest. 2022 Oct 17;132(20):e157975. doi: 10.1172/JCI157975 (PMC9566897; doi:10.1172/JCI157975)
Supplement: Supplemental data [file jci-132-157975-s048.pdf]

Supplemental Tables and Figures

Supplemental Table 1. Post-operative outcomes in the study cohorts

Supplemental Table 2. Univariate analysis to determine predictors of PGD.

Supplemental Figure 1. Deposition of LRA in native lungs and lung allografts. PE-labelled Col-V antibodies were injected in control wild-type mice or murine recipients 24 hours after lung transplant. Two photon imaging was performed during the described time-course. Magenta: LRA. Green: Blood vessels. Bar: 50 µm.

Supplemental Figure 2. Single Cell RNA sequencing from LRA and isotype control treated murine recipients. Mice in both groups were treated as described in the methods and then underwent syngeneic transplants. At 24 hours following transplantation, grafts were harvested and single cell RNA sequencing performed. The figure demonstrates the cell map.

Supplemental Figure 3. Anti-Ly6G antibody efficiently depleted neutrophils. WT recipient mice received 150 µg each (i.v.) of isotype control or LRA (collagen type V plus K-α1 tubulin) 24 hours before and 1 hour after lung transplantation as well as isotype or anti-Ly6G antibody 24 hours before lung transplant. (A) Histology showing neutrophil and mononuclear infiltration (arrowhead) in mice treated with isotype but not in anti-Ly6G antibody treated mice. (B) Quantification of neutrophil recruitment into lungs (neutrophils gating: live CD45+ SiglecF- CD11b+Ly6G+) (n = 3). Data are presented as mean ± SD. Graph was analyzed by Unpaired Student's t-test. **p<0.01. Bar: 20 µm.

Supplemental Figure 4. Chest radiograph and histology in a patient with pre-existing LRA who developed PGD. Both patients with pre-existing LRA who developed PGD showed signs of antibody mediated rejection. A representative patient is illustrated. A) Chest radiographs showing progressive PGD on post-operative days 1 (i) and 3 (ii) as well as improvement after 2 (iii) and 7 (iv) days of initiation of plasmapheresis and complement inhibition. B) Histological analysis of intraoperative post-reperfusion biopsy showed: (i) Lung parenchyma with fibrin, red blood cells and scattered neutrophils (20x), (ii) reactive alveolar pneumocytes with acute and chronic inflammatory cells (20x), (iii) capillary congestion (20x), and (iv) complement C4d staining. The donor lung prior to reperfusion appeared normal (v).

Supplemental Table 1. Post-operative outcomes in the LRA and non-LRA cohorts

Variable	Overall (n=56)	LRA (n=8)	Non LRA (n=48)	*P* value
PGD grade 3	4 (7.1%)	2 (25.0%)	2 (4.4%)	0.09
Ventilator use (days)	4.7 ± 7.3	3.8 ± 3.7	4.9 ± 9.9	0.77
ICU stay (days)	11.7 ± 11.0	9.2 ± 3.6	11.7 ± 11.8	0.54
Hospital stay (days)	21.6 ± 23.8	27.1 ± 40.5	20.7 ± 19.3	0.49

PGD, primary graft dysfunction; ICU, intensive care unit

Supplemental Table 2. Univariate Logistic Regression Analysis: Predictors of Postoperative Primary Graft Dysfunction

Variable	OR	*P* value	95% CI
LRA	7.33	0.06	0.87-62.2
Age, years	0.50	0.56	0.04-5.15
Female	0.45	0.49	0.04-4.35
BMI, kg/m2	1.14	0.38	0.84-1.54
BSA, m2	6.55	0.42	0.60-6.49
Smoking history	2.00	0.56	0.19-20.6
Hypertension	0.85	0.87	0.11-6.53
Diabetes	1.94	0.52	0.25-1.50
CKD	3.00	0.37	0.26-3.46
Panel Reactive Antibodies	1.47	0.28	0.58-2.73
Donor Specific Antibodies	1.22	0.72	0.46-1.62
Laboratory			
Hemoglobin, g/dL	0.98	0.72	0.65-1.34
WBC, 1,000/mm3	0.93	0.70	0.73-1.21
Platelets, 1,000/mm3	0.98	0.14	0.97-1.01
Sodium, mEq/L	0.94	0.81	0.61-1.25
BUN, mg/dL	0.88	0.33	0.67-1.14
Creatinine, mg/dL	3.01	0.40	0.04-8.66
ALT, U/L	0.98	0.70	0.89-1.07
AST, U/L	1.01	0.56	0.97-1.05
Albumin, g/dL	1.12	0.43	0.84-1.49
Total bilirubin, mg/dL	2.23	0.17	0.71-7.00
INR	0.73	0.89	0.01-5.93
ABG (at cannulation)			
pH	1.53	0.73	0.87-1.52
PaCO2	1.04	0.22	0.97-1.12
PaO2	0.99	0.60	0.98-1.00
HCO3	1.09	0.30	0.92-1.28
Donor			
Age, years	1.01	0.76	0.92-1.11
Female	0.42	0.47	0.04-4.36
Cause of death			
Head trauma	1.30	0.59	0.87-1.68
Drug overdose	1.06	0.98	0.78-1.34
Other	1.24	0.76	0.89-1.29

LRA, lung restricted antibody; BMI, body mass index; BSA, body surface area; CKD, chronic kidney disease; WBC, white blood cell; BUN, blood urea nitrogen; AST, aspartate aminotransferase; ALT, Alanine aminotransferase; INR, international normalized ratio

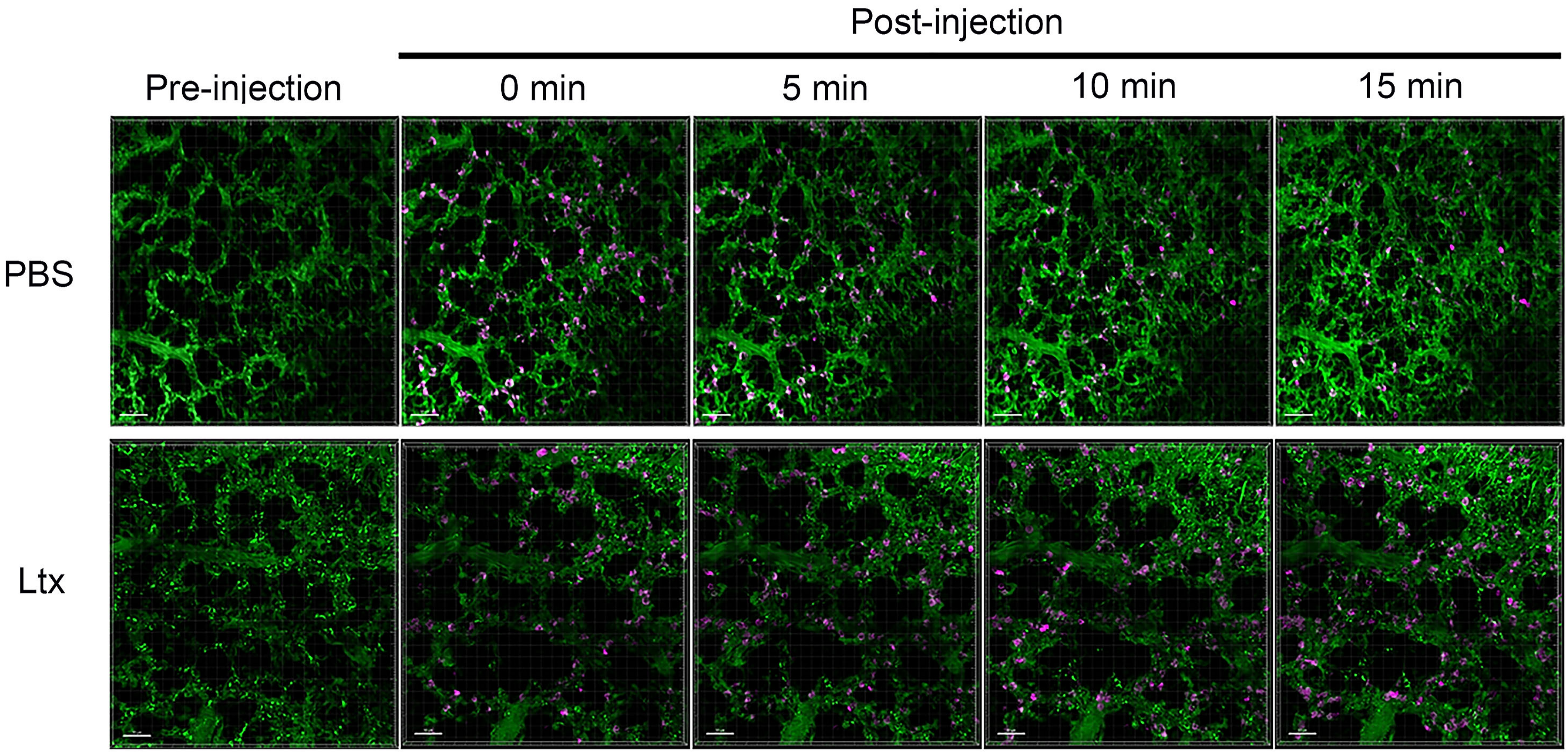

Supplemental Figure 1. Deposition of LRA in native lungs and lung allografts. PE-labelled Col-V antibodies were injected in control wild-type mice or murine recipients 24 hours after lung transplant. Two photon imaging was performed during the described time-course. Magenta: LRA. Green: Blood vessels. Bar: 50 μm.

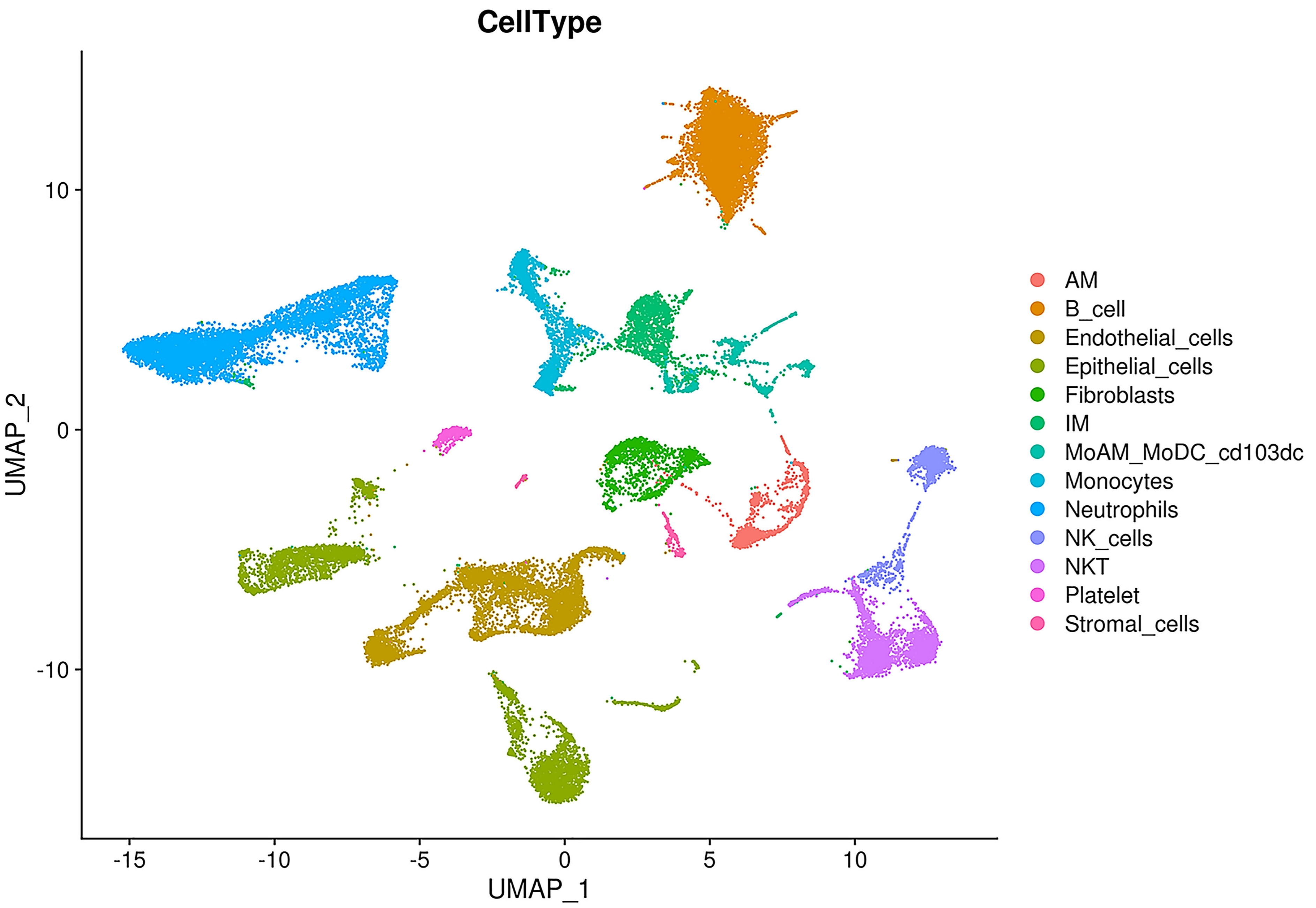

Supplemental Figure 2. Integrated single-cell RNA-Seq analysis of isotype or LRA-treated murine allograft after lung transplantation identifies diverse lung cell populations. Integrated single-cell RNA-Seq analysis of isotype and LRA-treat ed murine allograft after lung transplantation identifies diverse lung cell populations. Single-cell RNA-Seq was performed on single-cell suspensions generated from 2 allografts from isotype-treated and 2 from LRA-treated. All 4 samples w ere analyzed using the Seurat R package. Cells were clustered using a graph-based shared nearest neighbor clustering approach and visualized using a Uniform Manifold Approximation and Projection (UMAP) plot.

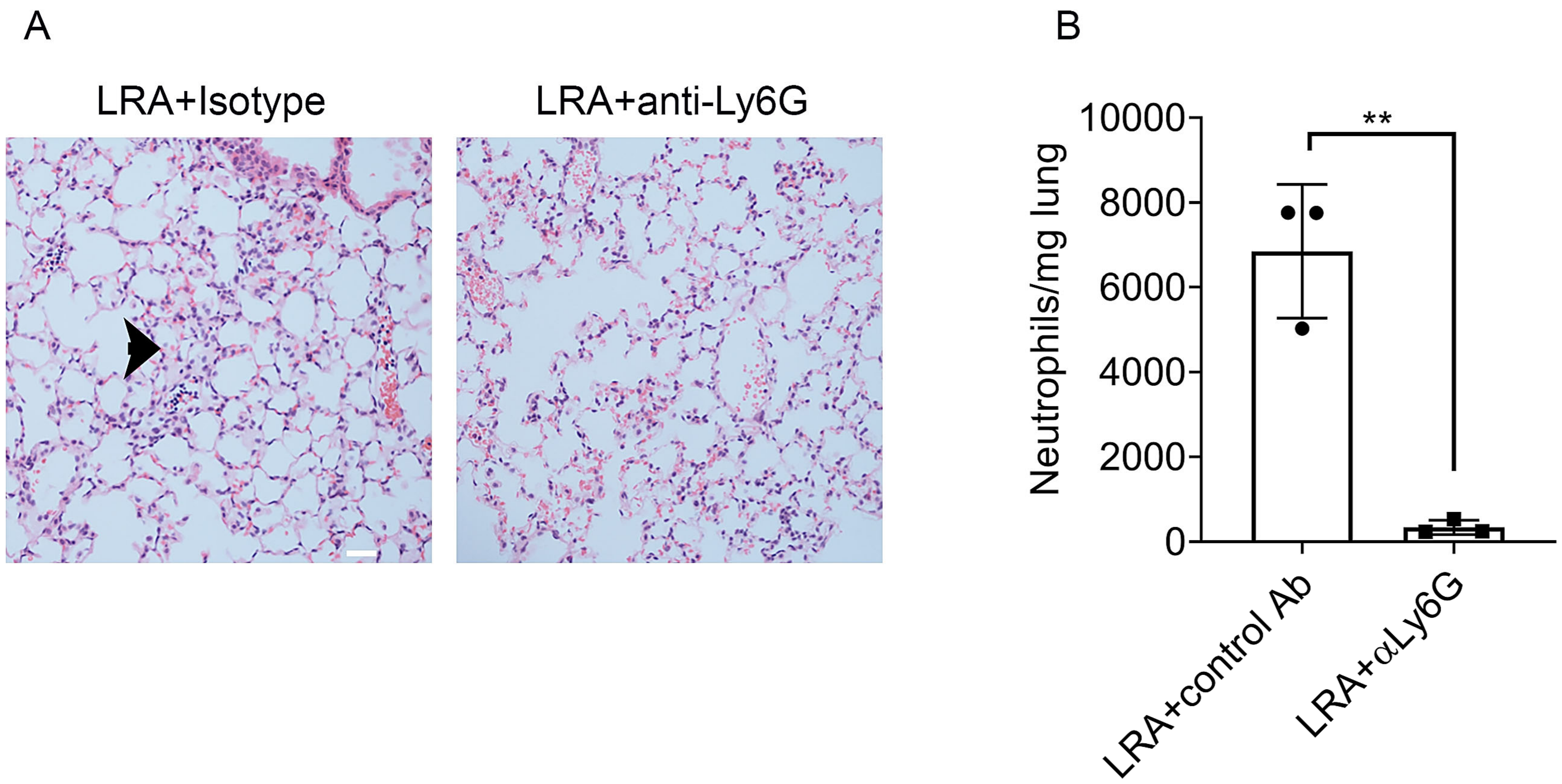

Supplemental Figure 3. Anti-Ly6G antibody efficiently depleted neutrophils. WT recipient mice received 150 µg each (i.v.) of isotype control or LRA (collagen type V plus K-α1 tubulin) 24 hours before and 1 hour after lung transplantation as well as isotype or anti-Ly6G antibody 24 hours before lung transplant. (A) Histology showing neutrophil and mononuclear infiltration (arrowhead) in mice treated with isotype but not in anti-Ly6G antibody treated mice. (B) Quantification of neutrophil recruitment into lungs (neutrophils gating: live $CD45^{+}$ $SiglecF^{-}$ $CD11b^{+}Ly6G^{+}$) (n = 3). Data are presented as mean ± SD. Graph was analyzed by Unpaired Student's t-test. **p<0.01. Bar: 20 µm.

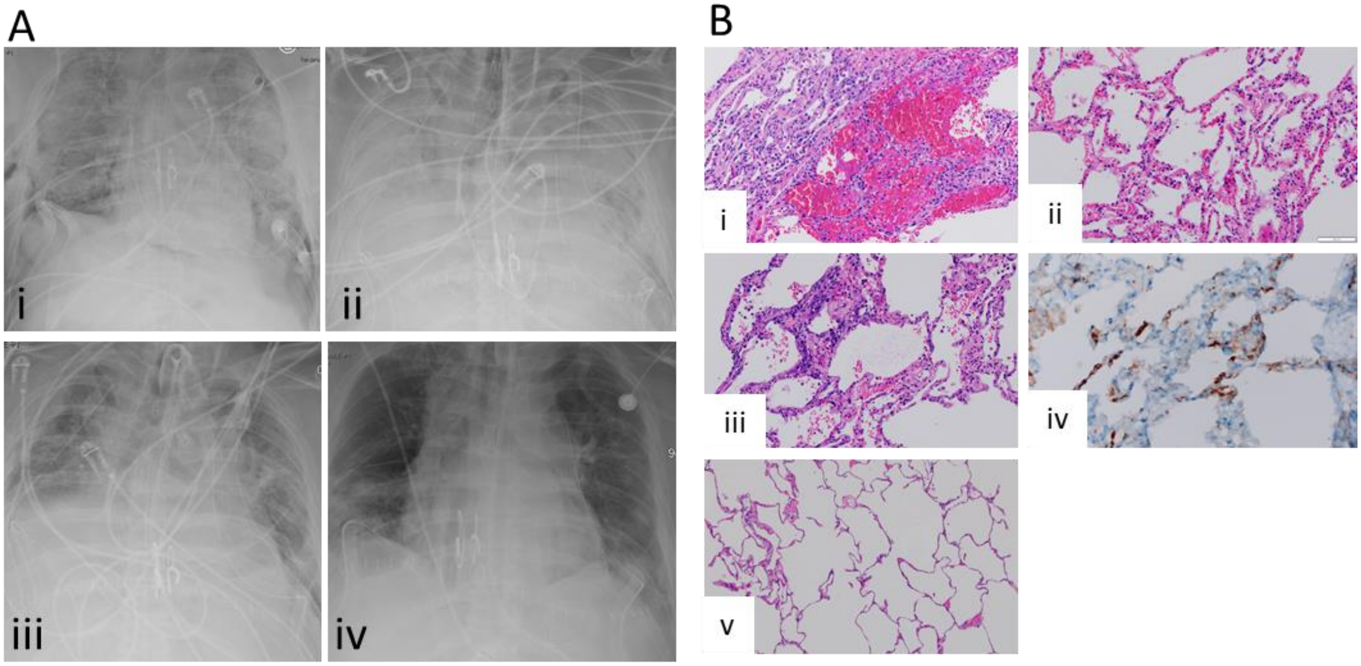

Supplemental Figure 4. Chest radiograph and histology in a patient with pre-existing LRA who developed PGD. Both patients with pre-existing LRA who developed PGD showed signs of antibody mediated rejection. A representative patient is illustrated. A) Chest radiographs showing progressive PGD on post-operative days 1 (i) and 3 (ii) as well as improvement after 2 (iii) and 7 (iv) days of initiation of plasmapheresis and complement inhibition. B) Histological analysis of intraoperative post-reperfusion biopsy showed: (i) Lung parenchyma with fibrin, red blood cells and scattered neutrophils (20x), (ii) reactive alveolar pneumocytes with acute and chronic inflammatory cells (20x), (iii) capillary congestion (20x), and (iv) complement C4d staining. The donor lung prior to reperfusion appeared normal (v).
